# Stress-response exhaustion in intervertebral disc degeneration

**DOI:** 10.3389/fcell.2026.1898234

**Published:** 2026-07-02

**Authors:** Jinghui Song, Tao Chen, Liang Ma

**Affiliations:** Orthopedics, Jingzhou Hospital Affiliated to Yangtze University, Jingzhou, China

**Keywords:** adaptive reserve, autophagy, cartilage endplate, cellular stress response, discogenic pain, intervertebral disc degeneration, senescence

## Abstract

Intervertebral disc degeneration (IVDD) is commonly framed as the cumulative result of extracellular matrix loss, inflammatory activation, oxidative damage, cellular senescence and cell death. This formulation is useful, but it also fragments the disease into parallel mechanisms and obscures a central paradox: nucleus pulposus and annulus fibrosus cells normally reside in a microenvironment that would be hostile to most mammalian cells. The healthy disc is avascular, diffusion limited, hypoxic, glycolytic, relatively acidic and mechanically loaded. These features are not simply pathological insults; they are defining ecological constraints to which disc cells are continuously adapted. Here we propose that IVDD can be productively reframed as a process of stress-response exhaustion. In this view, degeneration begins when the adaptive systems that maintain disc cell viability and matrix homeostasis under chronic microenvironmental stress lose amplitude, flexibility or recovery capacity. Hypoxia-inducible factor signalling, AMPK-mTOR metabolic sensing, autophagy and mitophagy, unfolded-protein and integrated stress responses, and redox buffering are initially protective. With ageing, endplate dysfunction, nutrient diffusion failure, acidosis, abnormal mechanical loading and organelle damage, these same systems can become insufficient or maladaptive, creating a degenerative tipping point. Downstream consequences include senescence, sterile inflammation, cell death, matrix collapse and neuroimmune conversion to painful disease. This framework does not replace established mechanisms of IVDD; rather, it orders them along a temporal axis from adaptation to exhaustion. It also suggests stage-specific therapeutic logic: preserve adaptive reserve early, prevent stress-response collapse, suppress senescence and sterile inflammation, and target neuroimmune sensitization in painful degeneration.

## Graphical Abstract

**Figure:**
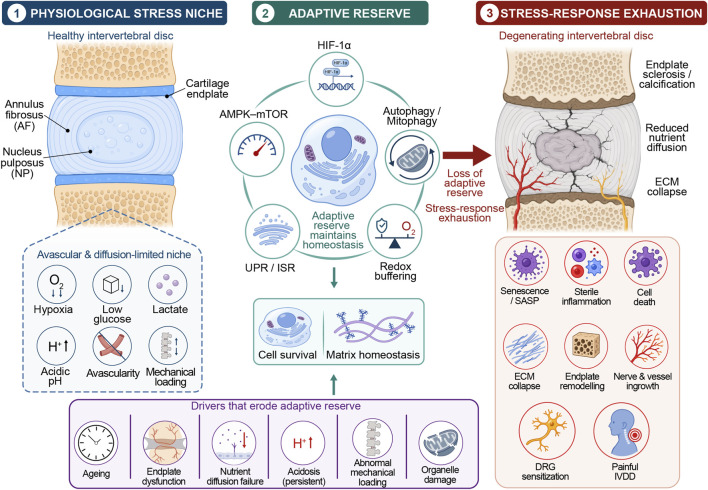

## Key points

- The intervertebral disc is a physiological stress niche rather than a conventional well-perfused tissue; hypoxia, nutrient limitation, lactate accumulation, acidic pH and mechanical loading are intrinsic features of disc biology.
- Disc degeneration can be reframed as a transition from stress-tolerant cellular adaptation to stress-exhausted degeneration, rather than as a simple accumulation of isolated molecular injuries.
- HIF-1α signalling, AMPK-mTOR sensing, autophagy, mitophagy, UPR/ISR and redox buffering are best interpreted as biphasic systems: protective when dynamic and recoverable, deleterious when chronically activated, blocked or exhausted.
- A degenerative tipping point emerges when endplate diffusion failure, acidosis, organelle stress and ECM-mechanical feedback exceed the cell’s adaptive reserve.
- Painful IVDD is likely a neuroimmune phenotype, not merely a more severe structural phenotype; future human studies should separate painful from non-painful degeneration.
- Therapeutic strategies should be matched to disease stage: adaptation support, exhaustion prevention, senescence-inflammatory control and pain-axis targeting.

## 1 Introduction: reframing IVDD as stress-response exhaustion

Low back pain remains one of the leading causes of disability worldwide, and degeneration of the intervertebral disc is one of its most intensively studied structural correlates (GBD 2021 Low Back Pain Collaborators, 2023; Urban and Roberts, 2003). During the past 3 decades, IVDD research has produced a rich mechanistic vocabulary: matrix catabolism, inflammatory cytokines, oxidative stress, endoplasmic reticulum stress, impaired autophagy, mitochondrial dysfunction, cellular senescence, apoptosis, pyroptosis, ferroptosis, angiogenesis, nerve ingrowth and dorsal-root-ganglion sensitization (Adams and Roughley, 2006; Risbud and Shapiro, 2014; Lyu et al., 2021; Mohd Isa et al., 2022; López-Otín et al., 2023; Hammoor et al., 2026). These mechanisms are real and clinically relevant. Yet, when arranged as a catalogue, they offer limited explanatory power. They tell us what changes in a degenerating disc, but less clearly explain why the same tissue can remain functionally stable for decades despite living under conditions that are, by design, metabolically austere and mechanically demanding.

This paradox is the starting point for the present Review. The nucleus pulposus (NP) and inner annulus fibrosus (AF) are not failed versions of highly vascularized tissues; they are specialized cellular ecosystems adapted to diffusion-limited nutrient supply, low oxygen tension, glycolytic metabolism, lactate production, acidic extracellular pH and fluctuating compressive and tensile loads (Holm et al., 1981; Grunhagen et al., 2006; Bibby and Urban, 2004; Bibby et al., 2005; Bartels et al., 1998). A healthy NP cell is therefore not merely tolerating stress. It is organized around stress. The disc microenvironment is hostile by design, and disc homeostasis depends on continuous adaptation to that hostility.

The central question is not simply what damages the disc, but when and why disc cells lose their ability to adapt to their native hostile microenvironment. We use the term stress-response exhaustion to describe the progressive loss of cellular adaptive reserve under chronic microenvironmental stress. The concept is not intended as a new molecular pathway or a substitute for established IVDD biology. Instead, it is an organizing principle. It emphasizes the transition from reversible adaptation to maladaptive persistence, from dynamic stress-response cycling to failed recovery, and from silent structural degeneration to inflammatory and painful phenotypes.

To keep this concept operational rather than rhetorical, we define stress-response exhaustion by a single unifying criterion: the loss of a disc cell’s capacity to restore homeostasis after a defined stress, as distinct from the mere presence of pathway activation. This loss of recovery capacity may be evidenced by one or more of the following: impaired recovery of adaptive signalling after challenge; blocked autophagic flux rather than altered static markers; persistent, unresolved ER stress; failed mitophagy with accumulation of damaged mitochondria; collapse of redox buffering; or a sustained senescence-associated secretory phenotype. These features are neither obligatory nor necessarily concurrent, and we do not claim that every degenerating disc follows one identical molecular sequence; the working definition and the measurable readouts that operationalize it are detailed in Box 1 and Box 3.

The stress-response-exhaustion framework has three advantages. First, it allows the same pathway to be interpreted differently across time. HIF-1α signalling, AMPK activation, autophagy, unfolded-protein responses and antioxidant programmes are not intrinsically good or bad; their consequences depend on duration, intensity, cellular age, nutrient context and recovery kinetics (Rajpurohit et al., 2002; Risbud et al., 2006; Agrawal et al., 2007; Risbud et al., 2010; Silagi et al., 2021; Madhu et al., 2020; Silagi et al., 2020; Silagi et al., 2018; Yurube et al., 2020). Second, it places the cartilage endplate and matrix mechanics upstream of many cellular events, because diffusion failure and altered loading determine whether intrinsic constraints remain physiological or become pathological (De Geer, 2018; Ren et al., 2023; Iatridis et al., 2006; Setton and Chen, 2006; Fearing et al., 2018; Wang et al., 2007). Third, it links structural degeneration to pain by positioning sterile inflammation, endplate remodelling, nerve ingrowth and neuroimmune sensitization as late consequences of an exhausted stress niche rather than simple markers of advanced matrix loss (Mohd Isa et al., 2022; Gilbert et al., 2016; Zhao et al., 2021; Aoki et al., 2014; Krock et al., 2014; Richardson et al., 2009; Sun et al., 2022).

Accordingly, the intended contribution of this Review is not to provide another comprehensive catalogue of IVDD pathways, which has been addressed in disease primers and mechanism-focused reviews (Risbud and Shapiro, 2014; Lyu et al., 2021; Hammoor et al., 2026). Its novelty lies in treating degeneration as a temporal and state-dependent loss of stress-response capacity: the same pathway can be protective during recoverable adaptation, maladaptive during chronic activation and insufficient once adaptive reserve is exhausted. This framing also separates structural degeneration from painful degeneration by requiring an additional neuroimmune and endplate-related conversion before imaging changes become clinically symptomatic.

### 1.1 Scope and evidence base

This is a conceptual, narrative Review rather than a systematic review. The literature was selected to support a mechanistic synthesis across disc biology, ageing, stress-response pathways, endplate transport, matrix mechanics and pain. Priority was given to peer-reviewed studies that provided direct evidence in intervertebral disc cells or tissue, human imaging or surgical samples, animal models with degenerative and pain-related outcomes, and widely accepted work from adjacent fields when disc-specific evidence was incomplete. The purpose of this approach is not to claim that stress-response exhaustion is a single measurable pathway, but to test whether established observations can be ordered into a temporal model that generates clearer experimental questions.

The framework should therefore be judged by its falsifiability and clinical usefulness. It would be weakened if longitudinal human or animal studies showed no relationship between declining endplate transport, impaired stress-response recovery kinetics, senescence-inflammatory conversion and pain-related phenotypes. It would be strengthened if stage-specific markers of adaptive reserve predicted progression or treatment response better than structural imaging grade alone.

To avoid overinterpreting a conceptual synthesis, the evidence base should be read through explicit levels of support. Throughout this Review, human tissue or imaging data are treated differently from animal-model evidence, cell-based mechanistic data and hypotheses extrapolated from adjacent stress-biology fields.

Box 1 **Working definition: stress-response exhaustion in IVDD**

Stress-response exhaustion denotes the state in which disc cells retain evidence of stress-pathway activation but lose the capacity to restore homeostasis after repeated or sustained challenge. It can manifest as reduced response amplitude, delayed recovery, chronic low-grade pathway activation, impaired flux through adaptive systems, failure to resolve organelle damage, metabolic inflexibility, senescence-associated secretory conversion or terminal stress-related cell death. In this Review, the term is used as a temporal and systems-level framework. It should not be interpreted as a single molecular biomarker, nor as a claim that every degenerating disc follows one identical pathway.

Box 2 **Evidence calibration for the stress-response-exhaustion framework.**

Box 3 **Operational features and measurable readouts of stress-response exhaustion**

Stress-response exhaustion should be operationalized as a loss of recovery capacity rather than as simple pathway activation. Useful readouts include: (1) microenvironmental transport and chemistry, including endplate permeability, glucose availability, lactate clearance and extracellular or intracellular pH; (2) adaptive signalling kinetics, including HIF-1α target genes, AMPK-mTOR activity and return to baseline after challenge; (3) quality-control flux, including autophagic flux rather than static LC3-II expression, mitophagy, lysosomal function, CHOP/XBP1 resolution and NRF2/GPX4 redox reserve; and (4) terminal conversion markers, including p16INK4a/p21, SASP cytokines, NF-κB/NLRP3 activation, NGF/CGRP expression and DRG or spinal sensitization. The framework would be weakened if these dynamic readouts did not outperform isolated endpoint markers in predicting progression, pain phenotype or treatment response.

## 2 The disc microenvironment: a physiological stress niche

The intervertebral disc is frequently described as poorly nourished, hypoxic and acidic, but these descriptors are sometimes presented as if they were purely pathological. In fact, they define normal disc biology. The central disc is among the largest avascular tissues in the human body. Its cells are separated from the nearest blood supply by matrix and cartilage endplate, and their survival depends on steep gradients of glucose, oxygen, lactate and pH (Holm et al., 1981; Grunhagen et al., 2006; Bibby and Urban, 2004; Bibby et al., 2005; Bartels et al., 1998). This arrangement imposes a distinctive biology: slow turnover, low cellularity, glycolytic energy production, high matrix osmolarity and dependence on diffusion rather than perfusion. Degeneration begins not because the disc becomes stressed, but because a physiological stress niche loses its capacity for regulated adaptation.

### 2.1 Avascularity and nutrient limitation

NP cells receive nutrients mainly through diffusion across the cartilage endplate and vertebral capillary network. Small solutes such as glucose and oxygen enter from vertebral-body blood vessels and move through the endplate and matrix, while lactate and other metabolites leave through the reverse route (Grunhagen et al., 2006; Bibby and Urban, 2004; Bibby et al., 2005; Bartels et al., 1998; De Geer, 2018). This architecture makes the disc vulnerable to small decreases in endplate permeability. Theoretical and experimental work indicates that modest reductions in exchange area can produce disproportionate changes in central glucose, lactate and pH, particularly when cellular demand is high or matrix transport is impaired (Bibby et al., 2005; Bartels et al., 1998; De Geer, 2018; Ren et al., 2023).

Nutrient limitation is therefore not a binary state. Mild limitation is a physiological constraint; severe or chronic limitation is a driver of adaptive failure. Glucose is especially important because disc cells rely heavily on glycolysis. *In vitro* studies show that low glucose and acidic pH can compromise disc-cell viability, matrix synthesis and metabolic activity, whereas low oxygen alone is often better tolerated (Bibby and Urban, 2004; Bibby et al., 2005). This distinction is crucial for the stress-exhaustion model: hypoxia is a native condition, but hypoxia combined with glucose restriction, lactate accumulation and impaired metabolite clearance creates a qualitatively different state.

### 2.2 Hypoxia and glycolytic dependence

Hypoxia has a privileged role in disc biology. Unlike many differentiated cells that stabilize hypoxia-inducible factors only when oxygen falls, NP cells can maintain HIF-1α under relatively normoxic culture conditions, a feature interpreted as a metabolic adaptation to the disc niche (Rajpurohit et al., 2002; Risbud et al., 2006). HIF-1α supports glycolytic gene expression, regulates aggrecan expression, influences cell survival and coordinates pH-regulatory programmes (Risbud et al., 2006; Agrawal et al., 2007; Risbud et al., 2010; Silagi et al., 2021; Madhu et al., 2020). In this context, low oxygen is not simply a noxious stimulus. It is part of the developmental and metabolic identity of the NP.

This point has practical importance. A review that equates hypoxia with degeneration risks misrepresenting the disc. The more relevant question is when hypoxic adaptation becomes coupled to insufficient substrate supply, impaired waste clearance and matrix failure. In an intact niche, glycolysis supplies energy and lactate efflux preserves intracellular pH (Silagi et al., 2020; Silagi et al., 2018). When endplate transport declines or matrix diffusion is compromised, glycolytic dependence can become a liability because the same metabolism that sustains cells also imposes acid load.

### 2.3 Lactate accumulation and acidic pH

Lactate is not merely a waste product. In the disc, it is an expected consequence of glycolytic life under low oxygen tension. Lactate efflux and bicarbonate recycling are therefore central to disc-cell viability (Silagi et al., 2020; Silagi et al., 2018). However, as permeability decreases and lactate removal fails, extracellular pH can fall to levels that suppress matrix synthesis, alter enzyme activity, promote catabolic signalling and sensitize acid-sensing ion channels (Bibby et al., 2005; Silagi et al., 2020; Gilbert et al., 2016). Acidic pH may also interact with inflammatory pathways and pain mechanisms, providing a bridge between metabolic exhaustion and nociceptive conversion (Gilbert et al., 2016; Zhao et al., 2021).

The emerging literature on lactate-derived histone lactylation adds another layer to this biology (Sun et al., 2025; Liu et al., 2025). It is tempting to make lactylation a central mechanistic axis, but at present it should be treated cautiously in IVDD. The more mature conclusion is that lactate has moved from being an inert metabolic endpoint to a signalling substrate. Within a stress-exhaustion framework, lactate is best viewed as a marker and mediator of the shift from adaptive glycolysis to maladaptive acid burden. Within the evidence calibration of this Review, lactylation is treated as a hypothesis-level mechanism rather than an established disc pathway (Box 2).

### 2.4 Mechanical loading

Mechanical loading is also biphasic. The disc requires physiological loading to maintain matrix turnover, solute transport and tissue-level function. Compression-relaxation cycles help drive fluid movement, whereas tensile and shear forces shape AF biology and cell-matrix interactions (Iatridis et al., 2006; Setton and Chen, 2006; Fearing et al., 2018; Wang et al., 2007). Disc cells respond to mechanical stimuli through calcium transients, cytoskeletal remodelling, integrin signalling, mechanosensitive channels and changes in gene expression (Fearing et al., 2018; Wang et al., 2007).

**FIGURE 1:**
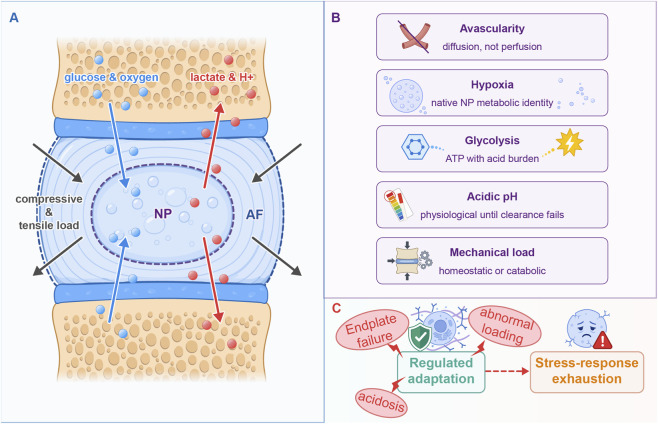
The disc as a physiological stress niche. **(A)** Glucose/oxygen diffuse into NP while lactate/protons are exported under compressive & tensile loading of disc and AF. **(B)** Five features: avascularity, hypoxia, glycolysis, acidic pH, mechanical load. **(C)** Flowchart linking adaptation to stress-response exhaustion (endplate failure, acidosis, abnormal loading). These features are physiological constraints rather than isolated pathological insults.

Abnormal loading changes this relationship. Excessive compression, torsion, repetitive vibration or altered segmental mechanics can amplify inflammation, matrix catabolism and cell death (Iatridis et al., 2006; Setton and Chen, 2006; Fearing et al., 2018; Wang et al., 2007; Boos et al., 2002). Importantly, mechanical stress should not be conceptualized as an independent insult that simply adds to metabolic stress. It modifies diffusion, fluid flow, matrix hydration, cell deformation and inflammatory signalling. As proteoglycans are lost and the matrix desiccates, cells experience a different mechanical microenvironment, which further disturbs metabolism and stress signalling. Thus, mechanical and metabolic stress form a coupled feedback system. The disc microenvironment is hostile by design; degeneration begins when this hostility exceeds the cell’s adaptive capacity. This physiological-to-pathological transition is summarized in Figure 1.

## 3 Cellular adaptation: survival strategies under chronic stress

If the disc is a physiological stress niche, its cellular pathways must be interpreted as survival strategies before they are interpreted as pathology. HIF-1α, AMPK-mTOR, autophagy, mitophagy, UPR/ISR and redox buffering are often discussed in IVDD as disease-associated mechanisms. Yet their primary biological purpose is to preserve homeostasis when nutrients fluctuate, proteins misfold, organelles are damaged or redox balance is disturbed. The shift from adaptation to exhaustion depends less on the mere presence of pathway activation than on its dynamics: whether the response is proportionate, whether damaged components are cleared, and whether the cell can return to a stable baseline after challenge.

### 3.1 HIF-1α-mediated hypoxic adaptation

HIF-1α is the most characteristic adaptive regulator of NP cells. Constitutive or normoxic stabilization of HIF-1α distinguishes NP cells from many other mesenchymal cell types and helps explain their glycolytic dependence (Rajpurohit et al., 2002; Risbud et al., 2006). HIF-1α regulates genes involved in glucose metabolism, pH regulation, matrix homeostasis and cell survival (Risbud et al., 2006; Agrawal et al., 2007; Risbud et al., 2010; Silagi et al., 2021; Madhu et al., 2020; Silagi et al., 2018). It is therefore misleading to treat HIF activation as intrinsically degenerative. In the normal disc, HIF signalling is a core element of niche fidelity.

At the same time, HIF-dependent adaptation has limits. If glucose availability drops, lactate efflux fails or intracellular pH regulation becomes insufficient, a HIF-driven glycolytic programme can no longer maintain homeostasis. The relevant disease question is not whether HIF is active, but whether the HIF programme remains matched to nutrient supply and metabolite clearance. Under stress-response exhaustion, a pathway originally designed for survival may persist in a microenvironment in which its outputs are no longer adequate.

### 3.2 AMPK-mTOR and metabolic sensing

AMPK and mTOR form a central axis for deciding whether cells conserve energy, degrade damaged components, synthesize matrix or enter stress-associated growth arrest. AMPK senses energetic stress and promotes catabolic, energy-sparing programmes, including autophagy. mTOR supports protein synthesis and cell growth when nutrients are sufficient (Yurube et al., 2020; Jiang et al., 2015). In the disc, this axis is particularly relevant because cells must balance the high biosynthetic demands of matrix maintenance against limited nutrient supply.

An adaptive AMPK response can restrain biosynthesis, promote repair and facilitate survival during transient stress. Conversely, persistent energetic insufficiency can impair matrix synthesis, alter autophagy kinetics and push cells toward senescence or death. mTOR suppression can be beneficial when it restores proteostatic balance, but excessive or chronic suppression may undermine the anabolic work needed to maintain aggrecan-rich matrix. The stress-exhaustion framework therefore interprets AMPK-mTOR not as a single therapeutic switch but as a rheostat whose optimal setting changes with disease stage.

### 3.3 Autophagy and mitophagy

Autophagy is a housekeeping and survival mechanism by which cells degrade damaged proteins and organelles and recycle substrates during nutrient limitation. In disc cells, autophagy has been implicated in adaptation to osmotic stress, hypoxia, oxidative injury and ageing (Yurube et al., 2020; Kang et al., 2019; Xie et al., 2019; Jiang et al., 2015; Choi et al., 2016). Mitophagy, including PINK1/Parkin- and HIF-1α-BNIP3-related pathways, limits the accumulation of damaged mitochondria and thereby restrains ROS production and inflammatory signalling (Madhu et al., 2020; Xie et al., 2019; Wang et al., 2018; Ma et al., 2022).

The most important methodological point is that autophagy must be assessed as flux, not as a static marker. LC3-II accumulation may indicate increased autophagosome formation, blocked degradation or both. Similarly, p62 accumulation can indicate impaired autophagic clearance rather than pathway activation. In early adaptation, autophagy and mitophagy preserve proteostasis and organelle quality. In exhaustion, lysosomal dysfunction, substrate overload and chronic stress can convert autophagy from a protective flux into a stalled process that marks failure of cellular clearance.

### 3.4 UPR/ISR and proteostasis

Disc cells synthesize and maintain an extensive extracellular matrix, which places sustained demand on protein folding and secretory pathways. The unfolded-protein response (UPR) and integrated stress response (ISR) provide adaptive mechanisms to reduce translation, increase chaperone capacity, enhance degradation of misfolded proteins and restore proteostasis. PERK-eIF2α-ATF4, IRE1α-XBP1 and ATF6 signalling can therefore be interpreted as early protective programmes rather than purely pathological cascades (Wen et al., 2021; Novais et al., 2021b; Luo et al., 2022; Huang et al., 2023).

The difficulty is that chronic ER stress changes the meaning of these pathways. When proteostatic load persists, adaptive signalling can give way to CHOP induction, JNK activation, inflammatory outputs and apoptosis (Wen et al., 2021; Novais et al., 2021b; Luo et al., 2022; Huang et al., 2023). Recent work further suggests that ER stress in NP cells is regulated by the hypoxic niche itself, reinforcing the idea that disc stress pathways are highly integrated rather than isolated modules (Novais et al., 2021b). In a stress-exhaustion framework, UPR and ISR become maladaptive not because they are activated, but because they fail to restore a functional proteostatic steady state.

### 3.5 Redox buffering

Reactive oxygen species are often described as damaging by-products, but low-level ROS also act as signalling molecules that regulate adaptation, matrix turnover and stress responses. The disc therefore requires redox buffering rather than absolute ROS elimination. NRF2, superoxide dismutases, glutathione metabolism, thioredoxin systems and lipid-peroxide detoxification pathways, including GPX4, define the cell’s antioxidant reserve (Tang et al., 2019; Suzuki et al., 2013; Forcina and Dixon, 2019; Fan et al., 2023).

NRF2 is particularly relevant because it couples oxidative stress to autophagy through the KEAP1-NRF2-p62 feedback loop and protects against experimental disc degeneration (Tang et al., 2019). When redox buffering fails, ROS can damage DNA, proteins, lipids and organelles; sensitize cells to ferroptosis; and amplify inflammatory signalling (Tang et al., 2019; Suzuki et al., 2013; Forcina and Dixon, 2019; Fan et al., 2023). Thus, ferroptosis is best placed downstream of redox-buffering failure, not as a stand-alone chapter detached from disc ecology. The key transition is from ROS as regulated signal to ROS as evidence of exhausted buffering capacity.

### 3.6 Hippo–YAP mechanotransduction

A further mechanosensitive module that follows the same biphasic logic is the Hippo–YAP/TAZ pathway. YAP and its paralogue TAZ are transcriptional co-activators that translate mechanical and architectural cues, such as substrate stiffness, cell shape, cytoskeletal tension and osmotic state, into programmes governing proliferation, survival and matrix production (Zheng-Wei et al., 2023). In nucleus pulposus cells, YAP activity is responsive to physiological loading and to the soft, hydrated matrix of the healthy disc, where it supports proliferation, anabolic gene expression and resistance to apoptosis (Zheng-Wei et al., 2023; Croft et al., 2021). Reported effects of YAP in disc cells are, however, strikingly inconsistent: some studies describe YAP as protective and declining with degeneration, whereas others couple YAP/TAZ activation under a stiffened matrix to catabolic signalling and even ferroptosis (Zheng-Wei et al., 2023; Ke et al., 2023).

**FIGURE 2:**
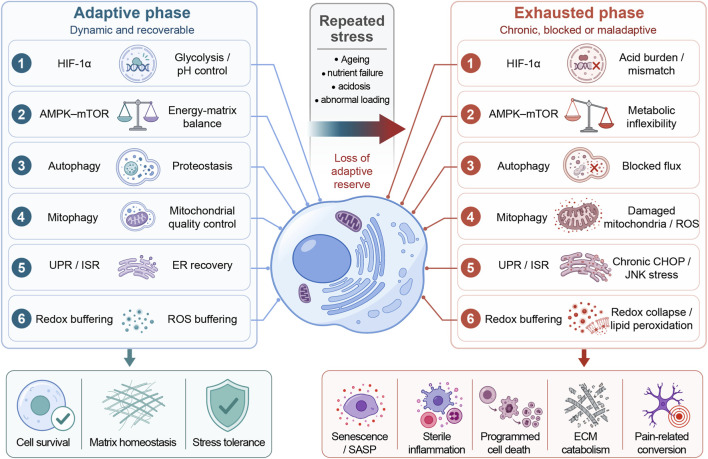
Protective adaptation *versus* maladaptive transition. HIF-1α, AMPK-mTOR, autophagy, mitophagy, UPR/ISR and redox buffering maintain disc-cell survival under native stress but become insufficient or maladaptive when stress burden exceeds adaptive reserve.

This apparent contradiction is exactly what a state-dependent framework predicts. Under physiological loading and an intact, compliant matrix, YAP/TAZ mechanotransduction is tuned to maintain proliferation and matrix anabolism; under abnormal loading, matrix stiffening and the inflammatory milieu of degeneration, sustained or dysregulated YAP/TAZ signalling instead couples to catabolic and cell-death programmes (Zheng-Wei et al., 2023; Ke et al., 2023). As with HIF-1α, AMPK-mTOR, autophagy and redox buffering, the meaningful question is therefore not whether YAP is intrinsically protective or harmful, but whether its mechanosensitive output remains matched to a physiological loading and matrix context (Sections 2.4 and 4.6). These biphasic stress-response modules are summarized in Figure 2 and Table 1.

**TABLE 1:** Biphasic actions of stress-response modules in intervertebral disc degeneration.

Stress-response module	Early adaptive role	Late maladaptive or exhausted state
HIF-1α	Maintains glycolytic metabolism, pH regulation, cell survival and selected ECM programmes in the hypoxic NP niche	Becomes insufficient when nutrient supply, lactate efflux and pH control fail; glycolysis contributes to acid burden
AMPK-mTOR	Matches energy status to biosynthesis, autophagy and substrate conservation	Metabolic inflexibility, impaired matrix synthesis, senescence-prone growth arrest or inappropriate anabolism under stress
Autophagy/mitophagy	Clears damaged proteins and organelles, limits ROS, supports survival during nutrient stress	Blocked flux, lysosomal dysfunction, p62 accumulation and failed removal of damaged mitochondria
UPR/ISR	Reduces translation load, promotes chaperone responses and restores proteostasis	Chronic ER stress, CHOP/JNK activation, inflammatory outputs and apoptosis
Redox buffering	Keeps ROS within a signalling range through NRF2, SOD, GSH and GPX4-related systems	Oxidative damage, lipid peroxidation, ferroptosis sensitivity and inflammatory amplification
Hippo–YAP/TAZ	Translates physiological loading and matrix compliance into proliferation, anabolic matrix gene expression and resistance to apoptosis	Under abnormal loading or matrix stiffening, sustained or dysregulated signalling drives catabolic and inflammatory programmes and ferroptosis
Senescence/SASP	May restrict propagation of damaged cells and provide transient repair signals	Persistent SASP, matrix catabolism, bystander dysfunction and chronic sterile inflammation

## 4 Degenerative tipping point: when adaptation becomes maladaptive

The most important implication of stress-response exhaustion is that degeneration is not simply a linear increase in injury. It is a phase transition. A degenerative tipping point occurs when adaptive responses can no longer restore matrix, metabolic and organelle homeostasis and instead become part of a self-amplifying disease loop.

### 4.1 Defining the degenerative tipping point

A tipping point can be conceptualized as the moment at which stress burden exceeds adaptive reserve. Stress burden includes nutrient limitation, oxygen gradients, lactate and proton accumulation, abnormal mechanical loading, inflammatory mediators, matrix fragments and organelle damage. Adaptive reserve includes HIF-dependent metabolic regulation, energy sensing, autophagic flux, mitophagy, proteostasis, antioxidant capacity, DNA repair and the ability to return to baseline after challenge. The transition from compensation to failure may be gradual in calendar time but abrupt in systems behaviour: once matrix hydration falls, endplate transport declines and inflammatory signalling increases, multiple feedback loops become mutually reinforcing.

This concept also explains heterogeneity. Two discs with similar imaging grades may differ markedly in stress reserve, cellular senescence, endplate permeability or inflammatory phenotype. Conversely, a disc with substantial structural degeneration may remain clinically silent if neuroimmune conversion has not occurred. The tipping point is therefore biological, not merely radiographic.

**FIGURE 3:**
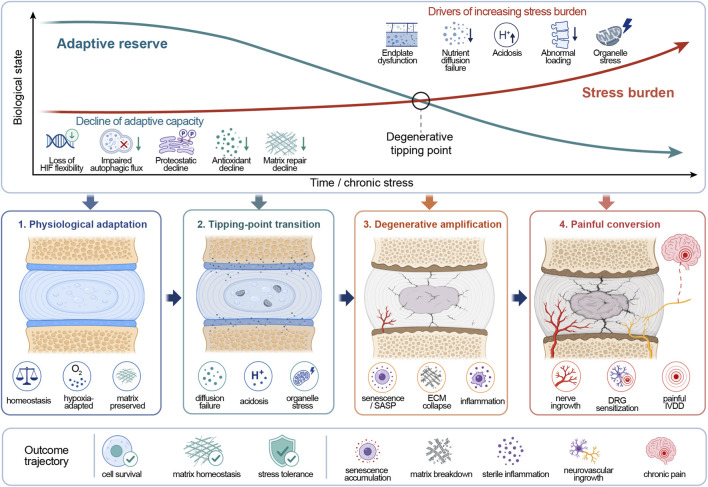
Degenerative tipping point model. Chronic ageing, endplate dysfunction and abnormal mechanics increase stress burden, while cellular adaptive reserve declines. Degeneration accelerates when the curves intersect.

The same framework accommodates the contrasting epidemiology of early-onset and age-related degeneration. Although advanced age is the dominant risk factor for disc degeneration, degeneration is increasingly observed in younger individuals, and the two groups need not share the same mechanism. In terms of the balance between stress burden and adaptive reserve, young-onset degeneration more often reflects an acute or repetitive rise in stress burden, such as mechanical overload, trauma, obesity-related loading or strong genetic predisposition, that transiently overwhelms an otherwise competent adaptive system. Age-related degeneration, by contrast, reflects a downward drift of adaptive reserve itself, through accumulated senescence, endplate calcification, declining autophagic and mitophagic flux and reduced redox capacity, so that even physiological stress can exceed reserve. In the terms of Figure 3, the former corresponds to an upward excursion of the stress-burden curve and the latter to a sagging of the reserve curve; both reach the same tipping point, but they imply different dominant biology and, potentially, different stage-appropriate interventions (Boos et al., 2002; Adams and Dolan, 2012; Patil et al., 2018).

### 4.2 Nutrient diffusion failure

Endplate dysfunction is one of the most plausible upstream drivers of stress-response exhaustion. Calcification, microdamage, sclerosis, inflammation and altered vascular supply reduce endplate permeability and disturb the balance between nutrient entry and metabolite exit (Grunhagen et al., 2006; Bartels et al., 1998; De Geer, 2018; Ren et al., 2023; Adams and Dolan, 2012). When diffusion is impaired, central disc cells experience lower glucose and oxygen and accumulate lactate and protons. Because the NP already operates near the edge of nutrient sufficiency, small transport deficits can have large effects.

The endplate can therefore be described as the metabolic gatekeeper of the disc. In early life, it enables the physiological stress niche to remain viable. With ageing and degeneration, it can become the structure through which physiological stress becomes pathological deprivation. This reframing has therapeutic implications: preserving or restoring endplate permeability may be as important as targeting NP cells directly.

### 4.3 Metabolic insufficiency and acidosis

Glycolysis is adaptive when substrate supply and lactate efflux are adequate. It becomes maladaptive when glucose is insufficient, ATP generation is limited, NAD+/NADH balance is disturbed and lactate clearance fails. Acidic pH suppresses matrix production, promotes catabolic enzymes, impairs cell viability and activates acid-sensing ion channels (Bibby and Urban, 2004; Bibby et al., 2005; Silagi et al., 2020; Gilbert et al., 2016; Zhao et al., 2021). It also alters inflammatory tone and may contribute directly to nociceptive sensitization.

Metabolic insufficiency should not be reduced to ATP depletion alone. The more relevant property is metabolic flexibility: the ability to shift substrate use, preserve redox balance, buffer pH and sustain matrix synthesis under fluctuating stress. Disc cells have limited flexibility because of their specialized hypoxic and glycolytic identity. As a result, the loss of one support system, such as lactate efflux or endplate glucose delivery, can destabilize multiple adaptive programmes simultaneously.

### 4.4 Organelle stress and redox collapse

Mitochondria in disc cells are not simply power plants; they participate in ROS generation, calcium handling, innate immune signalling, apoptosis and stress-response integration. Even in glycolytic NP cells, mitochondrial quality control matters. Damaged mitochondria can amplify oxidative stress and promote senescence, pyroptosis and apoptosis (Wang et al., 2018; Ma et al., 2022; Wen et al., 2021; Roughley, 2004). ER stress, mitochondrial stress and lysosomal dysfunction are likewise interconnected. Misfolded proteins increase ER load; impaired ER-mitochondria communication perturbs calcium and redox balance; blocked lysosomal degradation prevents removal of damaged organelles; and accumulating ROS further damages proteins and membranes.

For this reason, IVDD should not be written as a simple mitochondrial disease or a simple ER-stress disease. A more accurate model is an organelle-stress amplification network. The transition to redox collapse occurs when organelle damage exceeds the clearing capacity of mitophagy, ER-phagy, antioxidant programmes and proteasomal or lysosomal degradation. At this point, stress pathways cease to be reversible repair systems and become chronic inflammatory and death-promoting signals.

A specific subcellular structure helps explain why these organelle stresses tend to fail together rather than independently. Mitochondria-associated endoplasmic reticulum membranes (MAMs) are the contact sites at which the ER and mitochondria are physically and functionally coupled, coordinating Ca2+ transfer from ER to mitochondria, lipid exchange, mitochondrial bioenergetics and the priming of apoptosis. MAMs thereby act as a platform that integrates ER stress, redox signalling and mitochondrial quality control, and their disruption can translate ER stress into mitochondrial Ca2+ overload, ROS generation and apoptotic or inflammatory commitment (Yao et al., 2024). This provides a structural basis for the organelle-stress amplification network proposed here. Emerging disc-specific evidence supports this link: MAM disruption has been observed in degenerated nucleus pulposus, and restoration of MAM integrity, for example, through SYNJ2BP-facilitated ER-mitochondria contact, mitigates nucleus pulposus cell injury and experimental disc degeneration (Yao et al., 2024; Song et al., 2024). The MAM interface is thus a plausible node at which reversible organelle stress becomes self-amplifying.

### 4.5 Autophagy exhaustion and chronic ER stress

Autophagy exhaustion is not the absence of autophagy markers. It is the failure of autophagic flux to complete the work of cellular clearance. In ageing or chronically stressed discs, autophagosome formation, lysosomal function and substrate degradation can become uncoupled (Yurube et al., 2020; Kang et al., 2019; Xie et al., 2019; Jiang et al., 2015; Choi et al., 2016). This distinction is essential because therapies that merely increase autophagosome formation may not restore flux if lysosomal degradation remains impaired.

Similarly, chronic ER stress represents a failure of proteostatic adaptation. In early stress, PERK, IRE1α and ATF6 reduce folding burden and promote recovery. Under persistent overload, CHOP, JNK and inflammatory outputs connect ER stress to apoptosis, catabolism and senescence (Wen et al., 2021; Novais et al., 2021b; Luo et al., 2022; Huang et al., 2023). The interface between autophagy and ER stress is therefore a central component of the degenerative tipping point. Together, they determine whether damaged proteins and organelles are cleared or whether the cell enters a persistent state of maladaptive stress signalling.

### 4.6 ECM collapse and mechanical feedback

Matrix failure is both a consequence and a driver of stress-response exhaustion. Loss of aggrecan and type II collagen, together with increased MMPs and ADAMTS activity, reduces hydration, alters load distribution and exposes cells to abnormal mechanical stimuli (Risbud and Shapiro, 2014; Le Maitre et al., 2007; Zhao et al., 2011; Yurube et al., 2014; Zhang et al., 2022; Krock et al., 2017). Matrix fragments can also behave as damage-associated molecular patterns that activate toll-like receptors and inflammatory pathways (Krock et al., 2017). Thus, ECM breakdown is not a passive endpoint; it is an active source of cellular stress.

This mechanical-biochemical feedback loop helps explain why late-stage degeneration becomes self-sustaining. Reduced proteoglycan content decreases swelling pressure and changes mechanical strain. Altered strain increases catabolic gene expression and inflammatory signalling. Inflammation increases matrix degradation and cellular stress. Diffusion becomes more heterogeneous as structure fails. The disc then moves from an adaptive niche to a degenerative ecosystem. The stress-reserve tipping-point model is summarized in Figure 3.

## 5 Consequences of stress-response exhaustion

Once adaptive capacity is exhausted, the disc does not follow a single terminal pathway. Instead, it enters a set of overlapping outcomes: senescence, programmed cell death, sterile inflammation, endplate remodelling, angiogenesis, nerve ingrowth and pain.

### 5.1 Cellular senescence and SASP

Cellular senescence is a stable stress-associated growth arrest characterized by p16INK4a, p21, p53 activation, DNA-damage responses, chromatin remodelling and a senescence-associated secretory phenotype (SASP) (Patil et al., 2019; Novais et al., 2021a; Patil et al., 2018; Feng et al., 2016; He and Sharpless, 2017). In the disc, senescent cells accumulate with age and degeneration, and senescence has been linked to oxidative stress, inflammation, mechanical stress and impaired matrix synthesis (Patil et al., 2019; Patil et al., 2018).

Senescence is not necessarily harmful at inception. Transient senescence can restrain damaged cells and participate in tissue remodelling. The problem is persistence. Chronic SASP releases IL-6, IL-8, chemokines, matrix-degrading enzymes and growth factors that spread dysfunction to neighbouring cells and create a local inflammatory state (Patil et al., 2018; Feng et al., 2016; He and Sharpless, 2017). Experimental clearance of p16INK4a-positive senescent cells or senolytic treatment with dasatinib and quercetin ameliorates age-related disc degeneration in mice, providing some of the strongest causal evidence that senescence is not merely a marker of IVDD but a functional amplifier (Patil et al., 2019; Novais et al., 2021a).

### 5.2 Programmed cell death as terminal stress failure

Cell death pathways represent terminal modes of stress failure. Apoptosis reduces viable cell number and matrix maintenance capacity. Pyroptosis couples cell death to inflammatory cytokine release through inflammasome activation, caspase-1 and gasdermin-D-mediated membrane pore formation (Zhao et al., 2021; Ge et al., 2022). Ferroptosis reflects failure of iron and lipid-peroxide control, particularly within the GPX4-glutathione axis (Crump et al., 2023). These mechanisms overlap with the stress modules discussed above: redox collapse sensitizes cells to ferroptosis, mitochondrial damage primes apoptosis and inflammasome activation, and ER stress can promote both apoptosis and inflammatory death.

PANoptosis and other forms of integrated cell death may become relevant as the field matures, but they should not dominate the conceptual architecture of a review unless strong disc-specific evidence is available (Box 2). The broader point is that programmed cell death is not the origin of IVDD; it is often the endpoint of unresolved microenvironmental and organelle stress. Treating it effectively may require intervention before the cell has crossed the threshold from adaptive strain to terminal failure.

### 5.3 Sterile inflammation and immune activation

The healthy disc is relatively immune privileged, but degeneration disrupts structural and biochemical barriers. Stressed disc cells release inflammatory mediators, SASP factors and matrix fragments; AF tears and endplate damage permit vascular and immune-cell access; and damage-associated molecular patterns activate TLRs, NF-κB and NLRP3 signalling (Risbud and Shapiro, 2014; Lyu et al., 2021; Mohd Isa et al., 2022; Ge et al., 2022; Krock et al., 2017; Koroth et al., 2023; Dou et al., 2025). Inflammation in IVDD is therefore not simply imported from infiltrating immune cells. It is also produced by resident disc cells that have entered a state of stress-inflammatory conversion.

Macrophages and other immune cells can accelerate matrix degradation, vascular invasion and nociceptive signalling, but their roles are likely context dependent (Koroth et al., 2023; Dou et al., 2025). Early immune responses might clear debris and support repair, whereas chronic activation can maintain pain and degeneration. A stress-exhaustion view places sterile inflammation downstream of failed cellular adaptation and barrier breakdown, while acknowledging that inflammation then feeds back to worsen metabolism, matrix loss and pain.

### 5.4 Endplate and microenvironment remodelling

The endplate occupies a privileged position in this framework because it regulates nutrient access, metabolite removal, mechanical coupling and pain-related changes in the vertebral marrow compartment. Endplate calcification and sclerosis reduce diffusion; microfracture and inflammatory remodelling can produce Modic-like changes; and vascular and neural ingrowth alter the immune and sensory ecology of the motion segment (De Geer, 2018; Ren et al., 2023; Viswanathan et al., 2020; Conger et al., 2022; Wang et al., 2023a; Crump et al., 2023).

Endplate remodelling also challenges the tendency to study the NP in isolation. *In vivo*, the degenerating disc is part of a disc-endplate-vertebral-body unit. A biologically meaningful model of stress-response exhaustion must therefore include not only NP and AF cells but also cartilage endplate cells, subchondral bone, marrow inflammation, vascular channels and sensory innervation. The painful degenerative disc is not merely a collapsed matrix; it is a remodelled microenvironment.

## 6 Painful degenerative transition and therapeutic implications

A high-impact framework for IVDD must explain more than degeneration. It must explain why degeneration is painful in some individuals and clinically silent in others. Imaging studies in asymptomatic populations show that disc degeneration, bulging, protrusion and annular fissures are common and increase with age (Brinjikji et al., 2015). Structural degeneration is therefore neither necessary nor sufficient as a simple explanation of pain. Painful IVDD is better considered a neuroimmune phenotype superimposed on structural and cellular degeneration.

### 6.1 Why not all degenerated discs are painful

The mismatch between imaging and symptoms has two implications. First, human studies should distinguish painful from non-painful degeneration whenever possible, rather than treating surgical disc tissue as a uniform disease state. Second, animal models should incorporate pain-related behaviours, sensory neuron changes or neuroimmune readouts, not only histological degeneration. Without this phenotyping, therapies may improve matrix appearance while failing to address pain.

Clinical phenotyping should therefore specify the pain construct being studied. Structural degeneration can be graded radiographically, for example, by Pfirrmann MRI grade (Pfirrmann et al., 2001), but this is not equivalent to axial discogenic pain. Annular fissure or high-intensity zone (HIZ), Modic/endplate change, vertebrogenic pain, radicular pain from nerve-root compression and mixed nonspecific chronic low back pain should not be pooled without justification (Viswanathan et al., 2020; Conger et al., 2022; Wang et al., 2023a; Brinjikji et al., 2015; Aprill and Bogduk, 1992). Provocative discography may enrich for painful internal disc disruption, but it remains controversial because the procedure is invasive and 10-year matched-cohort data associated discography exposure with accelerated degenerative changes (Carragee et al., 2009). These distinctions matter because an intervention aimed at endplate-related nociception, such as basivertebral nerve ablation, tests a different clinical phenotype than a strategy aimed at NP matrix regeneration or annular inflammation (Conger et al., 2022; Fischgrund et al., 2018). In this Review, therefore, discogenic (annular or internal disc disruption), vertebrogenic (endplate or Modic, basivertebral nerve), radicular (nerve-root) and nonspecific chronic low back pain are treated as distinct constructs with distinct mechanisms and targets, and are not pooled when pain-related evidence is interpreted.

A stress-response-exhaustion model predicts that pain emerges when matrix breakdown and endplate remodelling intersect with inflammatory mediators, vascular access, nerve ingrowth and sensitization of dorsal-root-ganglion (DRG) neurons. In other words, pain requires a transition in tissue ecology: the disc must become permissive to nociceptive signalling and neuroimmune amplification.

### 6.2 Neuroimmune mechanisms of painful IVDD

Inflammatory cytokines such as TNF, IL-1β, IL-6, PGE2 and CCL2 contribute to catabolism, immune-cell recruitment and nociceptor sensitization (Risbud and Shapiro, 2014; Lyu et al., 2021; Mohd Isa et al., 2022; Koroth et al., 2023; Dou et al., 2025). Nerve growth factor (NGF), brain-derived neurotrophic factor, substance P and calcitonin gene-related peptide (CGRP) connect degenerating disc tissue to sensory sprouting and dorsal-root-ganglion activation (Aoki et al., 2014; Krock et al., 2014; Richardson et al., 2009; Sun et al., 2022; Orita et al., 2010; Johnson et al., 2001; Koerner et al., 2016; Kartha et al., 2014). Painful human discs show increased expression of neurotrophic and nociceptive mediators, and experimental inhibition of NGF-related signalling can reduce CGRP expression in disc-innervating DRG neurons (Aoki et al., 2014; Krock et al., 2014; Richardson et al., 2009; Sun et al., 2022; Orita et al., 2010).

**FIGURE 4:**
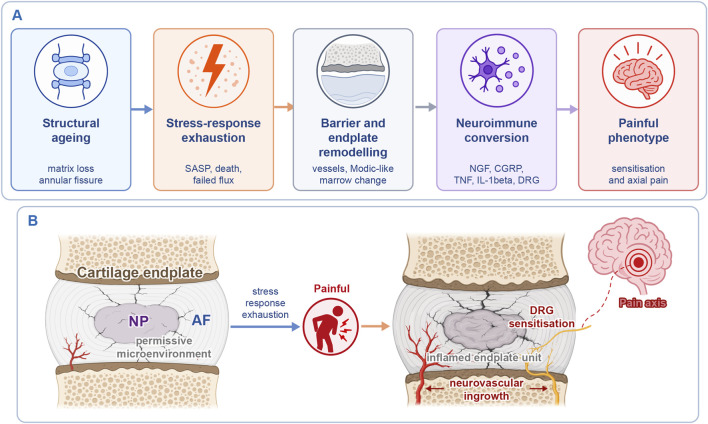
From silent degeneration to painful degeneration. **(A)** Five-step sequence: structural aging, stress-response exhaustion, barrier/endplate remodelling, neuroimmune conversion, painful phenotype. **(B)** Anatomical view of neurovascular ingrowth, endplate inflammation, DRG sensitization and pain-axis signalling to the brain.

Vascular and nerve ingrowth further distinguish painful degeneration from silent structural ageing. Matrix disruption and inflammatory signalling can permit blood vessels and nerves to penetrate regions that are normally aneural or sparsely innervated (Mohd Isa et al., 2022; Aoki et al., 2014; Krock et al., 2014; Richardson et al., 2009; Sun et al., 2022; Johnson et al., 2001). Endplate-driven pain adds another dimension. Modic changes and basivertebral nerve-related vertebrogenic pain indicate that the disc-endplate-vertebral body unit can generate axial pain through marrow inflammation and endplate injury, not solely through annular nociceptors (Viswanathan et al., 2020; Conger et al., 2022; Wang et al., 2023a). The proposed transition from silent degeneration to a painful phenotype is summarized in Figure 4.

### 6.3 Stage-specific therapeutic strategies

Therapeutic discussion in IVDD often becomes a list of molecules, biologics, biomaterials and cell therapies. A stress-exhaustion framework instead encourages stage matching (Table 2). In early adaptation, the goal is to preserve reserve: protect endplate transport, normalize mechanical loading, support NAD+ and mitochondrial quality, enhance appropriate NRF2 and autophagic responses, and avoid interventions that suppress physiological adaptation. During the tipping-point phase, the goal is to restore flux and prevent collapse: improve mitophagy, lysosomal function, ER proteostasis, pH handling and redox buffering. In the amplification phase, the goal shifts to senomorphic or senolytic strategies, inhibition of SASP, NF-κB or NLRP3, and control of matrix-degrading inflammation. In painful conversion, the target becomes neuroimmune signalling: NGF-TrkA, CGRP, TNF, IL-1β, DRG sensitization, vascular-neural ingrowth and endplate-related nociception (Lyu et al., 2021; Mohd Isa et al., 2022; Aoki et al., 2014; Krock et al., 2014; Richardson et al., 2009; Sun et al., 2022; Adams and Dolan, 2012; Roughley, 2004; Le Maitre et al., 2007; Zhao et al., 2011; Zhang et al., 2022; Lim et al., 2025).

**TABLE 2:** Stage-specific therapeutic logic derived from the stress-response-exhaustion model.

Disease stage	Dominant biology	Therapeutic goal	Candidate strategies
Early adaptation	Mild metabolic stress; preserved matrix; responsive HIF, AMPK, autophagy and redox programmes	Preserve adaptive reserve and endplate transport	Mechanical-load optimization, endplate protection, NRF2 support, NAD+/mitochondrial support, physiologic autophagy support
Tipping-point transition	Diffusion failure, acidosis, impaired autophagic flux, organelle stress and chronic UPR/ISR.	Prevent stress-response collapse	Restore autophagic flux, enhance mitophagy, improve pH handling, modulate ER stress, reduce oxidative overload
Degenerative amplification	Senescence, SASP, sterile inflammation, ECM catabolism and immune activation	Suppress inflammatory and catabolic amplification	Senolytics or senomorphics, SASP inhibition, NF-κB/NLRP3 modulation, anti-catabolic strategies
Painful conversion	Nerve and vessel ingrowth, DRG sensitization, Modic/endplate changes and neuroimmune signalling	Reduce nociceptive and neuroimmune amplification	NGF-TrkA, CGRP, TNF/IL-1β targeting, basivertebral/endplate pain strategies, phenotype-guided analgesic trials

This staged logic also clarifies why many promising interventions fail to translate. A treatment that enhances autophagy may help before irreversible lysosomal failure but be insufficient in a disc dominated by senescent SASP and nerve ingrowth. An anti-inflammatory agent may reduce pain while leaving metabolic exhaustion untouched. A cell therapy may fail if implanted into an acidic, nutrient-poor, mechanically unstable niche. The clinical question is not only which target is valid, but when the target is still reversible. It also follows that several of the strategies above, including NAD + support, senolytics and autophagy or mitophagy modulation, remain preclinical or hypothesis-generating rather than clinically established, whereas basivertebral nerve ablation and anti-NGF therapy already have human trial evidence in defined phenotypes, as set out in Table 3.

**TABLE 3:** Translational readiness of stage-specific therapeutic strategies.

Strategy	Most plausible stage or phenotype	Translational readiness	Main gating issue
Endplate transport and mechanical-load optimization	Early adaptation or tipping-point transition	Mechanistic and clinical rationale; needs imaging or transport biomarkers	Identify patients before irreversible matrix collapse
Autophagy, mitophagy and ER-stress modulation	Tipping-point transition	Mostly cell and animal evidence (Kang et al., 2019; Xie et al., 2019; Wang et al., 2018; Ma et al., 2022; Wen et al., 2021; Novais et al., 2021b; Luo et al., 2022; Huang et al., 2023)	Use flux-based assays and avoid static marker interpretation
Senolytics, senomorphics and SASP control	Degenerative amplification	Strong mouse causal evidence; human disc trials remain absent (Patil et al., 2019; Novais et al., 2021a)	Delivery, safety and identifying senescent-inflammatory discs
NAD+, NRF2 and redox-support strategies	Early adaptation or redox-vulnerable transition	Preclinical or extrapolated; NRF2 has disc-specific support (Tang et al., 2019)	Avoid treating oxidative stress as a uniform target.
NGF-TrkA, CGRP and inflammatory analgesic strategies	Painful neuroimmune conversion	NGF blockade has phase-3 CLBP evidence, but phenotype specificity and joint safety remain limiting (Orita et al., 2010; Markman et al., 2020)	Separate discogenic, vertebrogenic, radicular and nonspecific CLBP.
Basivertebral/endplate pain strategies	Modic or endplate-driven vertebrogenic phenotype	Randomized sham-controlled human evidence and clinical use in selected patients (Conger et al., 2022; Fischgrund et al., 2018)	Applies to endplate pain, not to all IVDD.
Cell, extracellular vesicle, biomaterial or regenerative therapy	Matrix failure with a repairable niche	Biologically plausible; translation depends on niche conditioning (Lim et al., 2025)	Acidic, nutrient-poor or mechanically unstable discs may reject repair

Thus, therapeutic claims should be calibrated by translational readiness rather than biological plausibility alone. Table 3 separates pathway support from clinical maturity and highlights the main gating risks that should determine whether a candidate is presented as preclinical, early human evidence, clinically used in selected phenotypes or still primarily hypothesis-generating.

### 6.4 Future directions

The field now requires temporal and spatial resolution. Single-cell and spatial transcriptomic studies have begun to map cellular heterogeneity in human discs, identify NP progenitor-like populations and reveal cell-cell communication during degeneration (Gan et al., 2021; Wang et al., 2023b; Liang et al., 2025; Stirnimann et al., 2025). The next step is not simply to produce more atlases, but to reconstruct disease trajectories. Which cell populations first lose adaptive reserve? Does endplate dysfunction precede NP senescence, or do they co-evolve? Which stress modules predict transition to pain? Which biomarkers distinguish adaptive stress from exhausted stress? Disease-trajectory studies should also stratify by age of onset, because young-onset and age-related degeneration may be dominated by different stress modules (rising stress burden *versus* declining adaptive reserve) and may therefore call for different stage-matched interventions.

Future models should include repeated-challenge experiments that measure response amplitude and recovery kinetics, not only endpoint expression. For example, disc cells from different degeneration grades could be exposed to standardized nutrient, acid, oxidative or mechanical stresses, followed by recovery assays of HIF signalling, autophagic flux, ER-stress resolution, mitochondrial quality and SASP. Such experiments would directly test whether degenerating cells have lost stress-response capacity rather than merely accumulated damage.

Clinical translation will require phenotyping. Human samples should record age, imaging grade, Modic status, pain phenotype, medications, comorbidities and tissue region. Animal models should measure pain-related behaviour and DRG/spinal sensitization alongside histology. Biomarkers should aim to stage discs as adaptive, tipping-point, amplification or painful-conversion states. The stress-response-exhaustion model will be useful only if it generates testable predictions (Box 4) and stage-specific therapeutic decisions.

Box 4 **Testable predictions generated by the framework.**

1. Dynamic challenge-recovery assays will separate adaptive from exhausted disc cells more clearly than endpoint expression of HIF, LC3, p62, CHOP, NRF2 or inflammatory markers alone.
2. Endplate diffusion impairment, acidosis and loss of autophagic/mitophagic flux should precede or accompany senescence-inflammatory conversion in longitudinal models.
3. Painful degeneration should show stronger coupling among SASP, sterile inflammation, vascular-neural ingrowth, endplate change and DRG sensitization than painless structural degeneration.
4. Stage-matched interventions should outperform pathway-focused treatments applied without biological staging.

**Table:** 

Evidence level	Examples within this Review	How conclusions are framed
Established in human tissue or imaging	Endplate transport constraints, disc hypoxia/acidosis, cytokine-rich painful discs, Modic/endplate changes, and the mismatch between structural degeneration and symptoms (Holm et al., 1981; Grunhagen et al., 2006; Bibby and Urban, 2004; Bibby et al., 2005; Bartels et al., 1998; Silagi et al., 2020; Silagi et al., 2018; Aoki et al., 2014; Krock et al., 2014; Richardson et al., 2009; Sun et al., 2022; Viswanathan et al., 2020; Conger et al., 2022; Wang et al., 2023a; Brinjikji et al., 2015)	Used to anchor the clinical relevance of the framework
Supported by animal models	Senescent-cell clearance, senolytic treatment, endplate microfracture models, mitophagy/autophagy modulation and DRG-related pain readouts (Wang et al., 2023a; Kang et al., 2019; Xie et al., 2019; Wang et al., 2018; Ma et al., 2022; Patil et al., 2019; Novais et al., 2021a; Orita et al., 2010)	Used as causal support, while acknowledging species and model limitations
Cell-based mechanistic evidence	HIF-1α signalling, AMPK-mTOR sensing, autophagic flux, UPR/ISR, NRF2/GPX4 redox buffering, inflammatory activation and cell-death modules (Rajpurohit et al., 2002; Risbud et al., 2006; Agrawal et al., 2007; Risbud et al., 2010; Silagi et al., 2021; Madhu et al., 2020; Silagi et al., 2020; Silagi et al., 2018; Yurube et al., 2020; Kang et al., 2019; Xie et al., 2019; Wang et al., 2018; Ma et al., 2022; Jiang et al., 2015; Choi et al., 2016; Wen et al., 2021; Novais et al., 2021b; Luo et al., 2022; Huang et al., 2023; Tang et al., 2019; Suzuki et al., 2013; Forcina and Dixon, 2019; Fan et al., 2023; Ge et al., 2022; Crump et al., 2023)	Used to define plausible mechanisms and dynamic readouts
Hypothesis or extrapolation	Lactylation-dominant mechanisms, PANoptosis, NAD + support and broad stage-matched treatment rules when direct disc-specific or human evidence is limited (López-Otín et al., 2023; Sun et al., 2025; Liu et al., 2025)	Presented as testable predictions rather than established therapeutic claims

## 7 Limitations of the framework

Several limitations should be made explicit to avoid overstatement. First, stress-response exhaustion is an integrative framework rather than a discrete pathway; it cannot be validated by a single biomarker. Second, much of the mechanistic evidence remains cell-based or animal-based, whereas human samples are often cross-sectional, regionally heterogeneous and biased toward surgical disease. Third, the same module may have opposite effects depending on degeneration stage, nutrient status, cell phenotype and recovery time; this complicates therapeutic translation. Finally, pain is not a direct readout of matrix degeneration. Any claim about painful IVDD should therefore require neuroimmune, endplate, vascular-neural or sensory-neuron evidence rather than imaging grade alone.

These limitations do not invalidate the model, but they define how it should be used. The most defensible version of the argument is not that every disc passes through a single universal sequence, but that degeneration becomes clinically important when adaptive reserve, matrix mechanics, endplate transport and neuroimmune signalling become coupled in a self-reinforcing state. This narrower claim is easier for reviewers to accept and easier for future studies to test.

## 8 Conclusion

IVDD can be understood as a progressive transition from stress-tolerant cellular adaptation to stress-exhausted degeneration. The healthy disc is not a benign environment that later becomes hostile; it is a physiological stress niche whose cells survive by continuously adapting to hypoxia, nutrient limitation, lactate accumulation, acidic pH and mechanical loading. HIF-1α signalling, glycolytic metabolism, AMPK-mTOR sensing, autophagy, mitophagy, UPR/ISR and redox buffering are therefore protective before they are pathological. Degeneration begins when endplate diffusion failure, acidosis, abnormal mechanical feedback and organelle damage exceed the capacity of these systems to restore homeostasis.

This framework does not deny the importance of ECM degradation, inflammation, senescence or cell death. Instead, it orders them. Matrix collapse, SASP, programmed cell death, sterile inflammation, endplate remodelling and pain are consequences of a niche that has lost adaptive reserve. The most useful future therapies may not be those that target a single pathway most potently, but those that intervene at the correct disease stage: preserving adaptation before exhaustion, restoring flux at the tipping point, suppressing senescence-inflammation during amplification and targeting neuroimmune mechanisms when degeneration becomes painful.
